# Microbiome dataset from the upper respiratory tract of patients living with HIV, HIV/TB and TB from Myanmar

**DOI:** 10.1016/j.dib.2018.10.003

**Published:** 2018-10-04

**Authors:** Kyaw Soe Htun, Yang Fong, Aye Aye Kyaw, Si Thu Aung, Khine Zaw Oo, Thein Zaw, Peter J. Lockhart, Bruce Russell, Gregory M. Cook, Htin Lin Aung, Tin Maung Hlaing

**Affiliations:** a Defence Services Medical Research Centre, Naypyitaw, Myanmar; b Institute of Fundamental Sciences, Massey University, Palmerston North, New Zealand; c National AIDS Programme, Ministry of Health and Sports, Naypyitaw, Myanmar; d National Tuberculosis Programme, Ministry of Health and Sports, Naypyitaw, Myanmar; e Department of Microbiology and Immunology, School of Biomedical Sciences, University of Otago, Dunedin, New Zealand

## Abstract

This article contains microbiome data from the upper respiratory tract of patients living with HIV/TB, HIV and TB from Meiktila, a town in Myanmar where there is a high incidence of HIV and TB. Microbiomes were compared for HIV/TB infected and healthy adults from the same population. We collected nasopharyngeal and oropharyngeal swabs from a total of 33 participants (Healthy {5}, HIV/TB {8}, HIV {14}, and TB {6}). DNA was extracted from the swabs and subjected to custom single step 16s rRNA sequencing on an Illumina MiSeq platform. The sequencing data is available via http://www.ncbi.nlm.nih.gov/bioproject/ PRJNA432583.

**Specifications table**

**Table:** 

Subject area	*Biology*
More specific subject area	*Microbiome, Infectious diseases*
Type of data	*Figure*
How data was acquired	*Culture-independent Illumina massively parallel sequencing of 16S rRNA genes using the Illumina sequencing-by-synthesis method on the MiSeq platform.*
Data format	*16S rRNA QIIME profiles*
Experimental factors	*Bacterial genomic DNA was extracted and used as a template to amplify the V3-V4 region of the 16S rRNA gene. The amplicons were barcoded, pooled, and sequenced using a paired-end protocol (Illumina).*
Experimental features	*Illumina massively parallel sequencing of the 16S rRNA gene libraries and OTU assignment analysis.*
Data source location	*Meiktila, Myanmar (Latitude: 20.8778 Longitude: 95.8584)*
Data accessibility	*Data is within this article and available via http://www.ncbi.nlm.nih.gov/ bioproject/ PRJNA432583*
Related research article	*Please add author names, title and publication details/status of the most relevant research article here, if available*

**Value of the data**

• These data are the first microbiome data reported from Myanmar. They provide insight into the microbial dynamics of infected individuals from a small population in a country where there is high prevalence of TB, drug-resistant TB and TB/HIV co-infection.
• Exploration of these data may contribute further insight into the prevention and treatment of bacterial infections in the respiratory tract of HIV, TB and HIV/TB patients, including the innovative use of probiotics.

## 1 Data

**Fig. 1:**
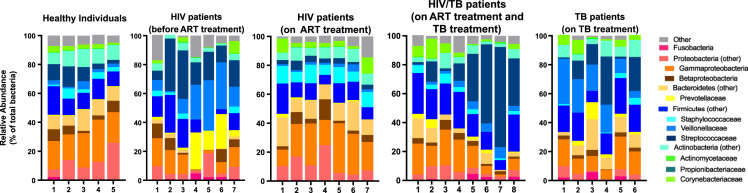
Relative abundance of operational taxonomic units (% OTU) indicating bacterial community composition of the upper respiratory tract of TB, HIV and HIV/TB patients and healthy individuals from Myanmar. The antiretroviral therapy (ART) treatment regimen contained Azidothymidine, Lamivudine and Efavirenz. The TB treatment regimen consisted of Isoniazid, Rifampicin, Streptomycin, Etambutol and Pyrazinamide.

16S rRNA profiles of infected adults were compared with those of healthy adults from the same population and small town in Myanmar. Fig. 1 shows the assignment of sequenced reads to operational taxonomic units and the bacterial community composition of the upper respiratory tract of TB, HIV and HIV/TB patients and healthy individuals.

## 2 Experimental design, materials and methods

We collected nasopharyngeal and oropharyngeal swabs from a total of 33 participants (Healthy {5}, HIV/TB {8}, HIV {14}, and TB {6}) living in Meiktila, a small town in Myanmar with a high incidence of HIV and TB. The swabs were collected by trained surveillance officers using internationally accepted Standard Operating Procedures (SOPs) as outlined in the Specimen Collection Guidelines from the CDC [1]. As per the Myanmar National AIDS Programme׳s Guidelines for Clinical Management of HIV Infection in Myanmar, HIV patients are on the antiretroviral (ART) treatment regimen containing azidothymidine, lamivudine and efavirenz and HIV/TB patients are on the ART regimen as well as the TB regimen containing isoniazid, rifampicin, streptomycin, etambutol and pyrazinamide [2]. Genomic DNA was extracted less than 24 h post-collection from each swab using the QIAamp DNA Microbiome Kit (Qiagen) as per the manufacturer׳s instructions. DNAs were then pooled and subjected to 16s rRNA sequencing.

For high-throughput 16s rRNA sequencing, a custom single-step dual-index PCR approach including the enrichment of the 16S rRNA V3 and V4 hypervariable regions was performed as described previously [3]. The development of each 16s rRNA V3-V4 forward and reverse primers consisted of Illumina adapters 29-nt forward sequence and 24-nt reverse sequence, an 8-nt index sequence, a 10-nt pad sequence, a 2-nt linker, and the gene-specific primer. The amplified products were then purified and normalized using SequelPrep™ normalization plate (ThermoFisher Scientific: Waltham, MA, USA), following the recommendations of the manufacturer. After normalization, 5 µl of each eluate was pooled together and quantified on a Qubit 3.0 Fluorometer (Life Technologies: Carlsbad, CA, USA) and Agilent Bioanalyzer High Sensitivity (HS) chip for visualization of 16S rRNA V3-V4 PCR band (600–650 bp) before dilution to 10 nM and 2 nM libraries. The prepared library was then sequenced as per manufacturer׳s protocol on a MiSeq for 500 sequencing cycles (2 × 250 PE) using version 2 chemistry and custom designed sequencing primers (for read 1, read 2 and index read) that targeted the 16S V3V4 region.

Image analysis, base-calling, raw data quality assessment and demultiplexing were processed on the MiSeq instrument via MiSeq Reporter version 2.6. Sequence trimming and analysis was conducted using Trimmomatic (v0.36) with a default parameter phred score of 33 [4]. Next the reads were merged into a single overlapping contig using FLASH, a paired-end assembler tool to improve assessment of bacterial diversity with overlapping reads of 100 bp before being processed via QIIME [5], [6]. For microbial profiling based on operational taxonomic unit (OTU) analysis, the UCHIME/UCLUST algorithm with “Greengenes” reference database was used to detect and remove any potential chimeric recombinant sequences from the generated data adjusted to 97% cutoff sequence similarity identity from the taxonomic classification of each read [7], [8]. Next, to assess whether microbial communities were significantly different UNIFRAC was used to estimate the overall phylogenetic distance between microbial communities from the generated OTU table [9].

The most abundant reads assigned to OTUs at phylum and family levels in HIV, TB, HIV/TB patients and healthy individuals are shown in Fig. 1. We observed decreased abundance of bacteria belonging to the family ‘Streptococcaceae’ and increased abundance of “Staphylococcaceae” in the upper respiratory tract of HIV patients on the ART regimen compared to HIV treatment naïve patients. However, the microbiota profile of HIV patients that are on the ART and co-infected with TB was similar to that of HIV treatment naïve patients except there was a lower abundance of “Veillonellaceae” in HIV/TB patients. The abundance of “Veillonellaceae” was higher in the TB patients on the TB treatment compared to the healthy individuals.
